# Serum glial fibrillary acidic protein predicts disease progression in multiple sclerosis

**DOI:** 10.1002/acn3.52187

**Published:** 2024-09-05

**Authors:** Evan Madill, Brian C. Healy, Negar Molazadeh, Mariann Polgar‐Turcsanyi, Bonnie I. Glanz, Howard L. Weiner, Tanuja Chitnis

**Affiliations:** ^1^ Brigham Multiple Sclerosis Center, Ann Romney Center for Neurologic Diseases Brigham and Women's Hospital Boston Massachusetts USA; ^2^ Harvard Medical School Boston Massachusetts USA

## Abstract

**Objective:**

Glial fibrillary acidic protein (GFAP) is expressed in astrocytes and may be a useful marker of non‐active progressive multiple sclerosis (MS). We evaluate serum GFAP (sGFAP) in a large cohort of MS patients to determine if it predicts progression independent of relapse activity (PIRA), future gait aid, and conversion to secondary progressive disease (SPMS).

**Methods:**

Adults with clinically isolated syndrome or any subtype of MS who were listed in the Brigham MS Center Research Database and had at least one sGFAP result were included. All clinic visits following first sample were analyzed for PIRA, future gait aid, and conversion to SPMS. Future cognitive dysfunction and fatigue were evaluated as secondary outcomes.

**Results:**

In total, 741 patients were included (average age: 42.3, average disease duration: 3.7 years, median EDSS: 2, and median follow‐up duration: 10.0 years). Of 643 patients (86.8%) without progressive disease at baseline, 15.9% developed SPMS. Among all 741, 50.5% had PIRA and 18.6% developed a gait aid requirement. sGFAP level predicted PIRA, future gait aid, and conversion to SPMS in univariable models (*p* < 0.001, <0.001, and 0.002). sGFAP remained predictive for PIRA and future gait aid in multivariable models in those younger than 50 (*p* = 0.048, 0.003). Change in sGFAP level over time was not predictive. There was no association between sGFAP and future fatigue or cognitive dysfunction.

**Interpretation:**

sGFAP helps to predict PIRA, future gait aid, and conversion to SPMS in a large cohort of MS patients. Our data suggest that baseline levels may be more useful than the change over time.

## Introduction

Multiple sclerosis (MS) is a heterogenous condition with a variable disease course. Approximately 15% of patients may have “benign MS,” with minimal disability decades after diagnosis, although estimates vary based on the definition that is used.^1^, ^2^ Many other patients develop progressive disease over a variable time course, with gradual worsening of imbalance, weakness, and other symptoms leading to significant disability. Although there are known predictors of disease outcomes at the population level, such as spinal cord atrophy and greater disability as measured by the expanded disability status scale (EDSS), prognostication for individual patients remains difficult.^3^, ^4^ With the emergence of highly effective therapies that have been shown to prevent conversion from relapsing disease and slow progressive MS, but that carry associated risks of immunosuppression, identifying patients at risk for disease progression has become particularly important.

Multiple serum biomarkers are now available for clinical use in the United States for patients with MS. Among these is glial fibrillary acidic protein (GFAP), a cytoskeleton protein that reflects astrocyte dysfunction and is associated with disease severity and disability progression in MS.^5^, ^6^ In particular, GFAP has been shown to predict confirmed disability progression among patients with low neurofilament light‐chain levels and more advanced disability.^7^, ^8^ As neurofilament light chain is associated with new gadolinium‐enhancing lesions and relapses, while GFAP is not significantly affected by new relapse activity, GFAP may be a better marker of progressive MS biology and could help predict disability progression in the absence of acute inflammatory lesions.^6^, ^9^, ^10^, ^11^ Stated another way, GFAP may help identify patients at risk of progression independent of relapse activity (PIRA), which can occur early in those with relapsing–remitting disease (RRMS) and is associated with a substantial risk of severe future disability.^12^ Despite the correlation between GFAP in cerebrospinal fluid and serum (sGFAP), other studies have found that cerebrospinal fluid levels, but not serum levels, are associated with confirmed disability progression.^13^, ^14^ The correlation of GFAP with brain atrophy also raises a question of whether GFAP could help to predict future cognitive impairment.^6^, ^15^ However, little has been published so far on any association with future cognitive dysfunction in MS or similar patient‐oriented outcomes such as fatigue.^16^, ^17^

In this study, we evaluate whether sGFAP is associated with future worsening across a large sample of patients with MS and varying degrees of baseline disability. Specifically, we investigate whether sGFAP helps to predict PIRA, conversion to secondary progressive MS (SPMS), and a future requirement for a cane or other gait aid. In a subset of patients, we explore whether sGFAP is associated with future fatigue or cognitive dysfunction as secondary outcomes.

## Methods

### Research ethics and informed consent

This study was approved by the Mass General Brigham Institutional Review Board under a secondary use protocol (#2024P000226). All patients consented to participation in previous biomarker studies at our center and the publication of anonymized results of subsequent retrospective analyses as secondary use.

### Inclusion criteria

Adult patients with clinically isolated syndrome (CIS) or any subtype of MS who were part of the Brigham MS Center Research Database and had at least one sGFAP result were included.

### Exclusion criteria

There were no prespecified exclusion criteria. Five patients were later excluded as outliers, as described in the results and Supplemental Materials.

### Data source

All patients had sGFAP levels drawn on at least one clinic visit date as part of prior biomarker studies at our center. These data were recorded along with demographic, clinical, and radiological information in the Brigham MS Center Research Database. The database includes information from each clinic visit (typically at 6‐month or yearly intervals), including the treating neurologist's assessment of EDSS and diagnosis, current disease‐modifying therapy, and whether interval clinical relapses or MRI lesions occurred. All clinic visits following first sample were analyzed for PIRA, new gait aid requirement, and conversion to SPMS. As part of prior studies, a subset of patients in our research database had longitudinal data available on fatigue (as assessed by the Modified Fatigue Impact Scale [MFIS]) and cognition (assessed by the Symbol Digit Modalities Test [SDMT]). The protocol for the administration of these assessments was previously published.^16^, ^18^

### Laboratory protocol

Serum was collected from patients in red‐top vacutainer tubes (glass, silicone coated, no additives). After 30–60 min at room temperature, samples were centrifuged for 10 min at 2000 revolutions‐per‐minute and 4°C. Tubes were then frozen at −80°C until analysis. sGFAP was quantified on a single molecule array platform from Quanterix (Billerica, MA) using the Neurology 4‐Plex A assay kit. Samples were measured over six runs using reagents from the same lot. Two quality controls provided with the kit were measured at the beginning and at the end of each run.

### Outcomes

Primary outcomes were PIRA, future gait aid requirement, and conversion to SPMS. We defined PIRA as an increase in EDSS of ≥1.5 among those with EDSS 0 at first sGFAP sample date (FSD), or ≥1.0 among those with EDSS 1.0–5.5 at FSD, or ≥0.5 among those EDSS 6.0 or higher at FSD. The change in EDSS was required to be sustained for at least 6 months and to occur in the absence of a clinical attack. Future gait aid requirement was recorded for patients with EDSS <6.0 at FSD who reached sustained EDSS 6.0 or greater. Conversion to SPMS was noted for patients with CIS or RRMS at FSD who were identified by their MS specialist to have SPMS at their most recent clinic visit. Among a subset of patients, we assessed future fatigue and cognitive dysfunction as secondary outcomes using total MFIS score and SDMT score.

### Statistical analysis

Multivariable linear regression was used to estimate the association between sGFAP level and age, disease duration, race, sex, smoking history, family history of MS, new clinical and MRI disease activity in the year prior to sample, EDSS, MS disease type, and disease‐modifying therapy at FSD to identify potential associations. To allow the results to be more easily applied by clinicians, we elected not to log transform sGFAP levels given the large sample size and exclusion of outliers.^19^ Similarly, we used unadjusted sGFAP levels, rather than *z*‐scores, as normative values for the Quanterix Neurology 4‐Plex assay kit are not currently available (to our knowledge). However, age and other variables relevant to sGFAP level, such as EDSS, were included in the multivariable analyses of disability worsening, as described below.

Comparisons of mean sGFAP level at FSD between those who did and did not reach the primary outcomes were done using t‐tests. Cox proportional hazards regression was performed for primary outcomes using patients' first sGFAP level and variables associated with sGFAP and the primary outcomes (age, sex, disease duration, EDSS, and MS subtype at FSD). Given the potential for sGFAP to increase for reasons other than MS in older adults, the increased likelihood of developing disability progression among those with known progressive disease, and our interest in predicting outcomes for young patients early in their disease course, we performed subgroup analyses on patients with CIS or RRMS who were younger than 50 years old at FSD.

For secondary outcomes, univariable linear regression was performed using sGFAP level at FSD and future SDMT and total MFIS scores. Age, sex, disease duration, EDSS, and MS subtype at fatigue/cognitive assessment date were also included in multivariable linear regression. We required that SDMT and MFIS assessments occur in the 2–5 years following FSD. This time period corresponded to the majority of the available data and was chosen to allow sufficient time for change to occur, while excluding a long tail of patients whose results might be influenced by much greater intervals between FSD and fatigue/cognitive assessments.

To investigate the utility of the change in sGFAP level over time, we again performed univariable and multivariable Cox proportional hazards regression using the difference between levels from the first and second sGFAP samples. Age, sex, disease duration, EDSS, and MS subtype at FSD were similarly included in the multivariable model. We required that the second sGFAP sample occur at least 6 months after FSD. Additionally, we excluded patients from this analysis if they reached the primary outcome before, or within 6 months of, the second sGFAP sample being drawn. Time to event or censoring was calculated from the date of the second sample rather than FSD in this case.

An alpha level of 0.05 was selected for all tests. We did not correct for multiple comparisons. All statistical tests were performed in StataSE (StataCorp, LLC., College Station, TX, United States).

## Results

### Patient characteristics

There were 741 patients included in our analysis (70.2% female). A median of 10.0 years of clinical follow‐up was available after FSD (interquartile range [IQR]: 6.0–12.7). The average age and median disease duration at FSD was 42.3 and 3.7 years, respectively (Table 1). A majority of patients had CIS or RRMS (86.8%). Among all patients, relatively few (12.4%) were on high‐efficacy therapy at FSD (defined as natalizumab, alemtuzumab, daclizumab, cladribine, cyclophosphamide, or any anti‐CD20 therapy). Approximately 2.5% were untreated at FSD. Five patients with sGFAP levels roughly ten‐fold higher than the study average (and almost double that of the next highest patients) were excluded as outliers (see Table S1 and Fig. S1).

**Table 1:** Patient characteristics.

Total number of patients	741
Age, years (mean, SD)	42.3 (11.7)
Sex (female)	520 (70.2%)
Race (White)	678 (91.6%)
Disease type	
CIS	39 (5.3%)
RRMS	604 (81.5%)
SPMS	60 (8.1%)
PPMS	38 (5.1%)
Disease duration, years (median, IQR)	3.7 (1.6–11.0)
EDSS (median, IQR)	2.0 (1.0–3.0)
DMT at sample draw	
High‐efficacy therapy	92 (12.4%)
Untreated	165 (22.3%)

Baseline characteristics of the study cohort at the time of first sGFAP draw (FSD). Abbreviations: SD – standard deviation; IQR – interquartile range; CIS – clinically isolated syndrome; RRMS – relapsing–remitting multiple sclerosis; SPMS – secondary progressive multiple sclerosis; PPMS – primary progressive multiple sclerosis; DMT – disease‐modifying therapy.

CIS, clinically isolated syndrome; DMT, disease‐modifying therapy; IQR, interquartile range; PPMS, primary progressive multiple sclerosis; RRMS, relapsing–remitting multiple sclerosis; SD, standard deviation; SPMS, secondary progressive multiple sclerosis.

### Associations with sGFAP

**Figure 1:**
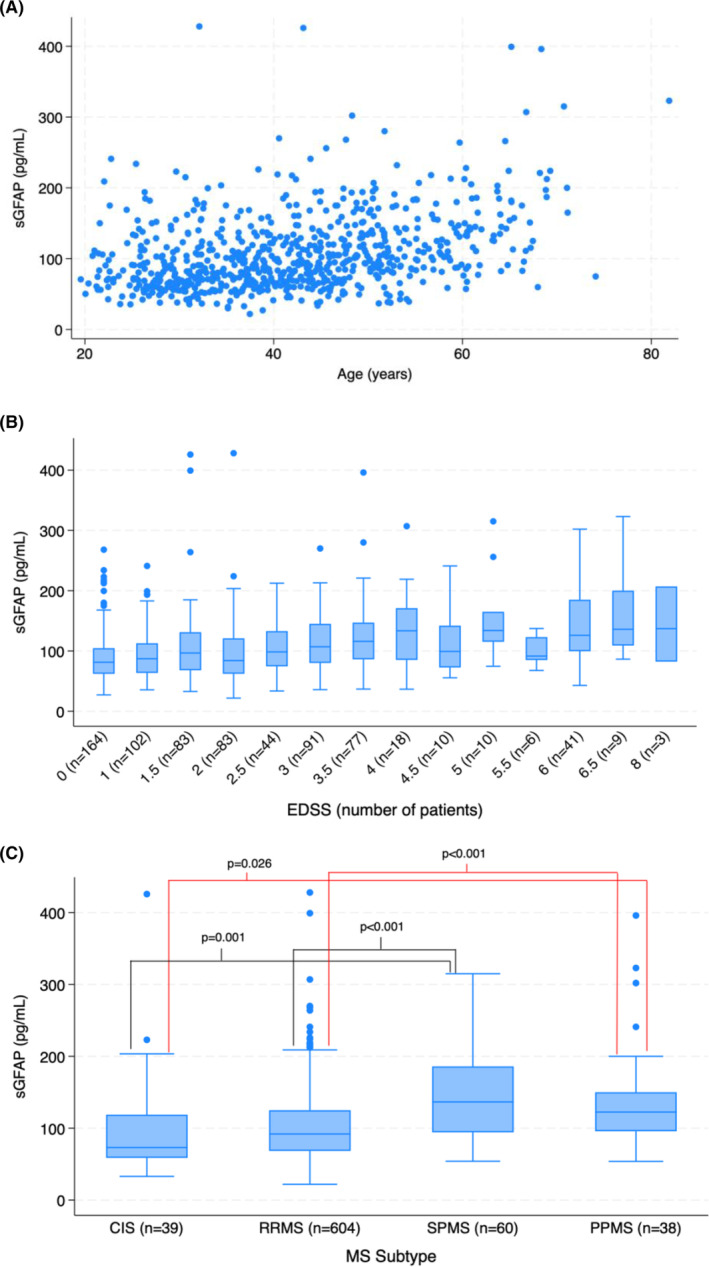
(A) Scatter plot showing an increase in sGFAP with increasing age (linear regression R‐squared: 0.125; *p* < 0.001), with a potential nonlinear increase after approximately age 50. (B) Box plot showing an overall increase in mean sGFAP level with increasing EDSS. The number of patients at each EDSS level at FSD is listed in parentheses. (C) Box plot of sGFAP level by MS subtype at FSD showing higher levels for progressive compared to nonprogressive patients. There was no significant difference between CIS and RRMS patients or between SPMS and PPMS patients.

Older age, female sex, longer disease duration, and higher EDSS were found to be significantly associated with higher sGFAP in a multivariable linear regression model (Table S2). There was a potential inflection point between ages 50 and 60, after which sGFAP began to increase non‐linearly (Fig. 1A). Mean sGFAP level was highest among patients with SPMS, followed by PPMS, then RRMS and CIS (146.0, 138.8, 102.0, and 101.2 pg/mL, respectively; Fig. 1C). Although sGFAP was higher among patients with progressive disease compared to CIS or RRMS, there was no significant difference between the SPMS and PPMS patients, or between the RRMS and CIS patients. There was a statistically significant difference between men and women, with sGFAP being about 10 pg/mL higher in women (Table S3). Notably, there was no association with high‐efficacy treatment or lack of treatment at FSD (Fig. S2). Patients with active MS (either clinical relapse or new MRI lesion) in the year before FSD had lower sGFAP levels than those without recent disease activity (97.1 vs. 115.3 pg/mL). However, this was confounded by older age, higher EDSS, and higher rates of progressive disease in the non‐active group; in multivariable regression, disease activity was not a predictor of sGFAP level. There was no difference in sGFAP level by race, smoking history, or family history of MS.

### sGFAP predicts future PIRA

**Figure 2:**
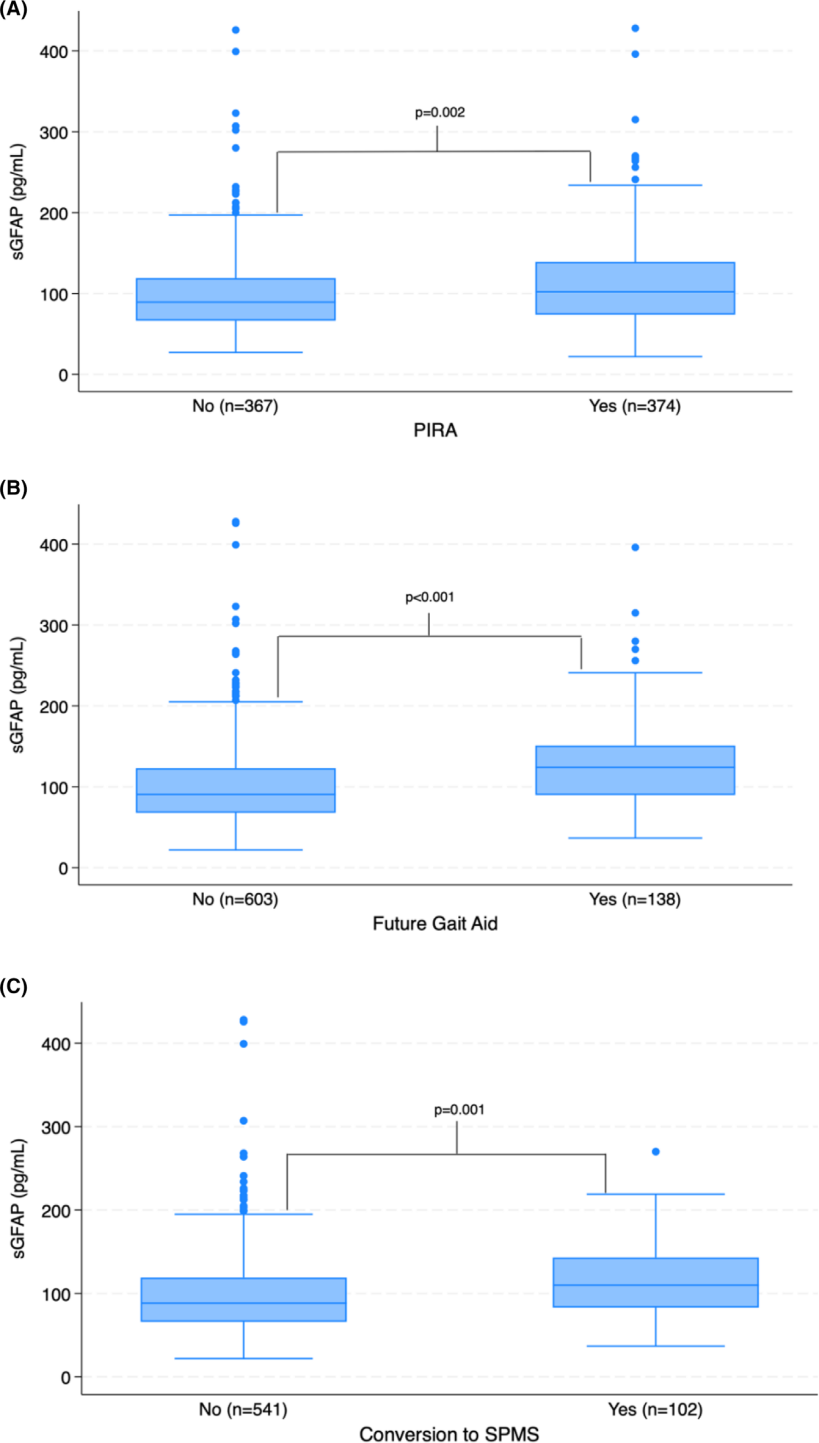
Box plots of sGFAP levels at FSD in patients with and without future PIRA (A), future gait aid requirement (B), and future conversion to SPMS (C).

**Figure 3:**
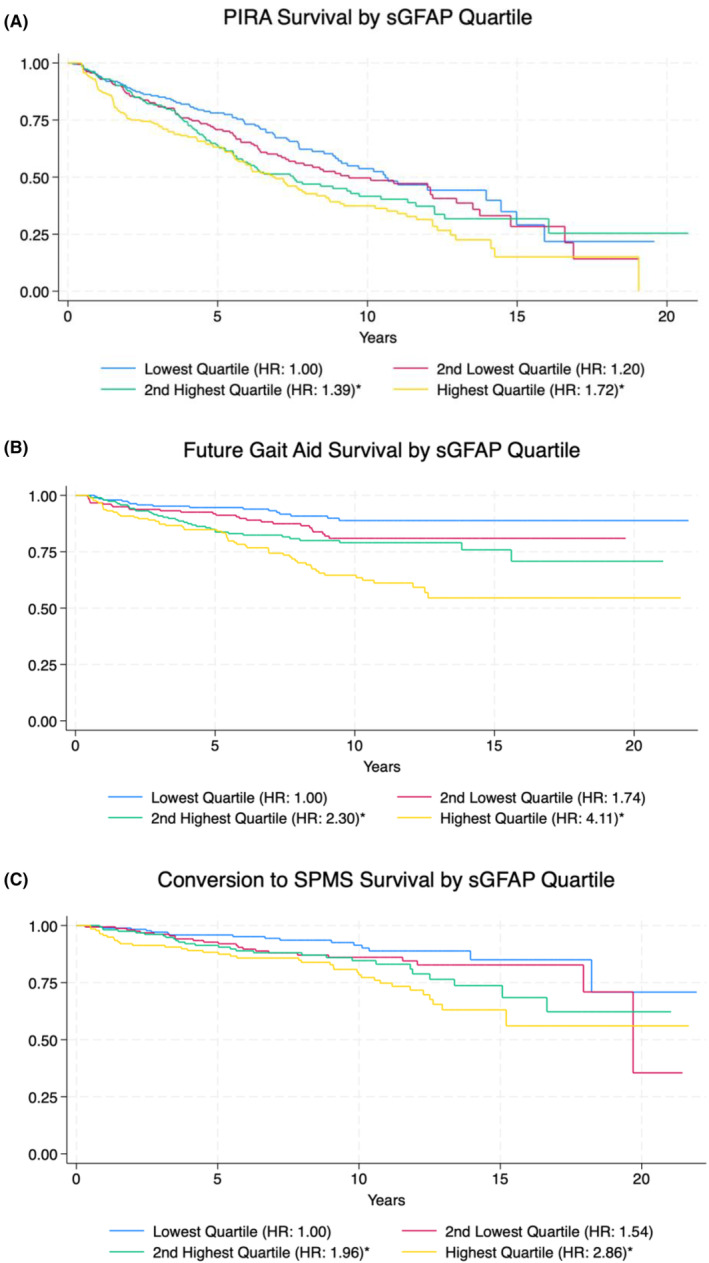
Kaplan–Meier curves showing time to PIRA (A), time to new gait aid (B), and time to SPMS conversion (C) by sGFAP level stratified by quartile. The lowest quartile corresponds to the 25% of patients with the lowest sGFAP level at FSD (blue). For each outcome, the HR of each quartile relative to the lowest quartile is included in parentheses in the legend (*p* < 0.05 is denoted by an asterisk). Fewer than 1% of patients remained at risk by year 20.

PIRA occurred in 374 (50.5%) of patients. Mean sGFAP level at FSD was higher among those with PIRA than those without (113.3 vs. 101.4 pg/mL) [Fig. 2]. Stratifying by quartile, time to PIRA was fastest in those in the highest quartile of sGFAP level (Fig. 3). Relative to the lowest quartile, the highest quartile of patients had a HR of 1.72 (95% confidence interval [CI]: 1.29–2.30; *p* < 0.001) for PIRA. In a univariable model (treating sGFAP level as a continuous variable), sGFAP was strongly associated with future PIRA with a hazard ratio (HR) of 1.032 per 10 pg/mL increase (CI: 1.015–1.050; *p* < 0.001) [Table 2]. After including age, EDSS, disease duration, sex, and MS subtype, sGFAP did not add significantly to the multivariable model (HR: 1.014; CI: 0.995–1.035; *p* = 0.157). As PIRA is more likely to occur in those with known progressive disease (68.4% of patients in our study, compared to 47.7% of those with CIS or RRMS at FSD), we analyzed a subset of patients to see if sGFAP could predict future PIRA among those with CIS/RRMS who were younger than 50. In this case, sGFAP provided additional predictive value holding age, sex, EDSS, and disease duration constant, conferring a 2.6% increased risk of PIRA for each 10 pg/mL increase in sGFAP (CI: 1.000–1.052; *p* = 0.048).

**Table 2:** Hazard ratios of primary outcomes by sGFAP level.

Outcome	Hazard ratio of univariable model [95% CI] (*p*‐value)	Hazard ratio of multivariable model [95% CI] (*p*‐value)
PIRA		
All patients (*n* = 741)	1.032 [1.015–1.050] (<0.001)	1.014 [0.995–1.035] (0.157)
CIS/RRMS and age < 50 (*n* = 515)	1.034 [1.010–1.058] (0.005)	1.026 [1.000–1.052] (0.048)
Future gait aid		
All patients (*n* = 741)	1.065 [1.042–1.089] (<0.001)	1.042 [1.012–1.073] (0.005)
CIS/RRMS and age < 50 (*n* = 515)	1.075 [1.045–1.107] (<0.001)	1.064 [1.022–1.108] (0.003)
Conversion to SPMS		
All CIS/RRMS (*n* = 643)	1.047 [1.016–1.079] (0.002)	1.013 [0.975–1.053] (0.510)
CIS/RRMS and age < 50 (*n* = 515)	1.059 [1.026–1.093] (<0.001)	1.037 [0.995–1.081] (0.088)

Values reflect the HR for each 10 pg/mL increase in sGFAP. The multivariable model includes age, EDSS, disease duration, sex, and MS subtype, in addition to sGFAP level at FSD, as inputs. For conversion to SPMS, only CIS and RRMS were included as categories in MS subtype, as patients with SPMS or PPMS at FSD were excluded from the analysis.

### sGFAP predicts future gait aid

A future gait aid requirement was seen in 138 of 741 patients (18.6%). Mean sGFAP level at FSD was higher among those who developed a future gait aid requirement (128.4 vs. 102.6 pg/mL) [Fig. 2]. Similar to PIRA, patients in the lowest quartile of sGFAP level had the slowest time to the development of a new gait aid (Fig. 3). Patients in the highest quartile of sGFAP at FSD had a HR of 4.11 relative to the lowest quartile (95% CI: 2.392–7.075; *p* < 0.001). Among all patients, higher sGFAP was a significant predictor of future gait aid requirement in both univariable and multivariable Cox proportional hazards models (Table 2). Holding age, EDSS, disease duration, sex, and MS subtype constant, each 10 pg/mL increase in sGFAP was associated with a HR of 1.042 (CI: 1.012–1.073; *p* = 0.005). To ensure that higher sGFAP did not primarily reflect the clinical course of progressive patients, we performed the same analysis among the 515 CIS/RRMS patients with age <50. sGFAP remained a significant independent predictor of developing a need for a gait aid among these patients, with a HR of 1.064 (CI: 1.022–1.108; *p* = 0.003) for each 10 pg/mL increase.

### sGFAP predicts conversion to SPMS but does not add to a multivariable model

Of the 643 patients with CIS or RRMS at FSD, 102 (15.9%) converted to SPMS. Mean sGFAP level at FSD was higher among those who converted to SPMS (117.0 vs. 99.1 pg/mL) [Fig. 2]. As with other primary outcomes, those RRMS/CIS patients in the highest quartile of sGFAP level were the most likely to convert to SPMS, with a HR of 2.86 (95% CI: 1.588–5.163; *p* < 0.001) relative to the lowest quartile (Fig. 3). Treated as a continuous variable, higher sGFAP level predicted conversion to SPMS in univariable analysis (HR: 1.047; CI: 1.016–1.079; *p* = 0.002), but not in a multivariable model with age, EDSS, disease duration, sex, and MS subtype (Table 2). The HR of sGFAP level was larger among patients younger than 50, but still did not contribute to a multivariable model of conversion (HR: 1.037; CI: 0.995–1.081; *p* = 0.088).

### sGFAP does not predict future cognitive dysfunction or fatigue

**Figure 4:**
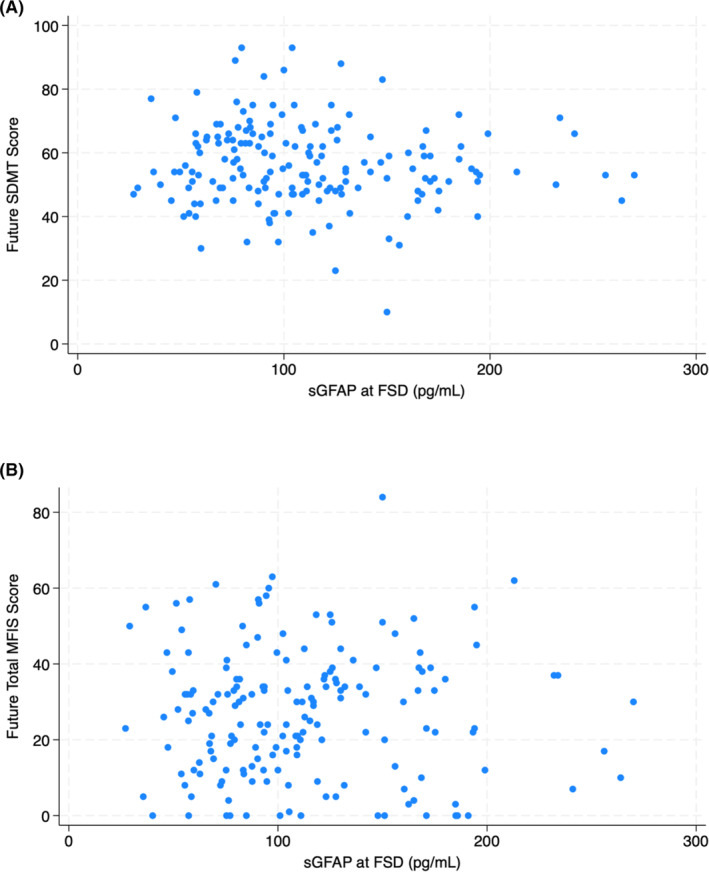
Scatter plots showing no association between sGFAP level at FSD and future SDMT score (A) and future total MFIS score (B). For the 173 patients included in this analysis, sGFAP levels were drawn 2–5 years prior to SDMT and MFIS assessments.

Among the 173 patients with an assessment 2–5 years after FSD, mean SDMT score was 56.1 (SD: 13.0) and mean total MFIS score was 26.8 (SD: 16.8) [Table 3]. The SDMT and MFIS were administered a median of 4.0 years after FSD (IQR: 3.1–4.2). There was no association with future SDMT score in univariable linear regression (coefficient: −0.027; 95% CI: −0.067 to 0.013; *p* = 0.183) [Fig. 4]. Similarly, sGFAP at FSD was not predictive of future SDMT score in multivariable linear regression holding age, EDSS, disease duration, sex, and MS subtype constant (coefficient: 0.004; 95% CI: −0.037 to 0.045; *p* = 0.850). There was also no association between sGFAP at FSD and future total MFIS score in univariable linear regression (coefficient: −0.006; 95% CI: −0.058 to 0.046; *p* = 0.830) nor in the multivariable model (coefficient: −0.030; 95% CI: −0.079 to 0.019; *p* = 0.227).

**Table 3:** Characteristics of patients with fatigue and cognitive assessments.

Total number of patients	173
Age, years (mean, SD)	46.3 (10.9)
Sex (female)	126 (72.8%)
Disease type	
CIS	3 (1.7%)
RRMS	137 (79.2%)
SPMS	25 (14.5%)
PPMS	8 (4.6%)
Disease duration, years (median, IQR)	8.0 (5.8–14.2)
EDSS (median, IQR)	1.5 (1.0–3.5)
SDMT score (mean, SD)	56.1 (13.0)
Total MFIS score (mean, SD)	26.8 (16.8)
sGFAP at FSD, pg/mL (mean, SD)	111.3 (48.7)
Time between FSD and SDMT/MFIS, years (median, IQR)	4.0 (3.1–4.2)

Characteristics of the subset of patients included in the fatigue and cognition secondary outcomes analysis. All data are reported from the time of SDMT/MFIS administration, except sGFAP level at FSD, which predates the fatigue and cognitive assessments by 2–5 years.

### Change in sGFAP over time is variable

A second sGFAP level was available at least 6 months after FSD in 653 (88.1%) patients. The median sample was drawn 1.3 years after FSD (IQR: 1.0–2.4). Over this time, sGFAP was minimally changed at a population level, but there was significant variability among individuals (mean change: −1.02 pg/mL, SD: 41.4 pg/mL). Age was correlated with the change in sGFAP level (linear regression coefficient: 0.275; *p* = 0.049), while higher baseline sGFAP was inversely correlated with the change over time (coefficient: −0.124; *p* < 0.001) in univariable linear regression. There was no association with EDSS, disease duration, sex, or MS subtype. sGFAP increased by 5.8 pg/mL (SD: 63.3) in 82 patients with a follow‐up sample who were on high‐efficacy therapy at FSD and by 7.0 pg/mL (SD: 30.2) in 16 untreated patients. There was no significant difference in the change in sGFAP level across any treatment status by ANOVA (*p* = 0.329).

### Change in sGFAP does not predict disability worsening

We found no association between change in sGFAP level and PIRA, the need for future gait aid, or conversion to SPMS in univariable or multivariable analysis (Table 4). However, sGFAP change did approach significance in a multivariable model of conversion to SPMS for CIS and RRMS patients younger than 50 (HR 1.120; CI: 0.999–1.256; *p* = 0.052). For these results, it should be noted that the sample sizes were smaller than those in the sGFAP level at FSD analyses, as some patients reached the relevant outcome prior to, or within 6 months of, the second sample being drawn.

**Table 4:** Hazard ratios of primary outcomes by change in sGFAP level.

Outcome	Hazard ratio of univariable model [95% CI] (*p*‐value)	Hazard ratio of multivariable model [95% CI] (*p*‐value)
PIRA		
All patients (*n* = 453)	1.018 [0.979–1.059] (0.376)	1.014 [0.973–1.058] (0.502)
CIS/RRMS and age < 50 (*n* = 310)	0.996 [0.923–1.075] (0.922)	1.006 [0.931–1.086] (0.883)
Future gait aid		
All patients (*n* = 572)	1.005 [0.951–1.062] (0.861)	0.981 [0.921–1.045] (0.550)
CIS/RRMS and age < 50 (*n* = 367)	1.023 [0.901–1.162] (0.725)	1.101 [0.969–1.252] (0.140)
Conversion to SPMS		
All CIS/RRMS (*n* = 589)	1.008 [0.950–1.069] (0.801)	1.021 [0.972–1.072] (0.408)
CIS/RRMS and age < 50 (*n* = 376)	1.046 [0.935–1.171] (0.432)	1.120 [0.999–1.256] (0.052)

Values reflect the HR for each 10 pg/mL increase in sGFAP. The multivariable model includes age, EDSS, disease duration, sex, and MS subtype, in addition to the change in sGFAP level, as inputs.

## Discussion

In our analysis, sGFAP was strongly associated with an increased hazard of PIRA, future gait aid requirement, and conversion to SPMS in a large sample of patients. There was a substantial increase in the HR of developing PIRA, a future gait aid, and conversion to SPMS among those in the highest quartile of sGFAP levels relative to the lowest quartile. Even when accounting for known predictors of disability worsening in MS (such as higher EDSS and progressive disease status), sGFAP provided additional value in predicting whether patients, including younger patients with CIS or RRMS, would develop a future gait aid requirement.^4^, ^12^, ^20^

sGFAP also contributed to a multivariable model of future PIRA among younger CIS/RRMS patients. However, the *p*‐value of 0.048 would not have been significant had we corrected for multiple comparisons. We elected not to do this beforehand because we believe the outcomes are correlated (i.e., those who develop a gait aid requirement are also likely to experience PIRA), reducing the likelihood of observing a significant result simply by running multiple analyses. The importance of sGFAP in a multivariable model of PIRA should be viewed in this context.

Regardless of correction for multiple comparisons, sGFAP was strongly associated with all primary outcomes in univariable analyses. For outcomes such as conversion to SPMS, clinical features including EDSS remained the most significant predictors in a multivariable model. Nevertheless, sGFAP may, to some degree, provide a quantitative summary of various clinical and demographic characteristics that are interpreted qualitatively in the context of clinical experience. In this way, sGFAP levels could potentially provide a more easily interpretable measure of risk of disability progression for patients and clinicians with less familiarity treating MS.

The change in sGFAP level over time was less helpful than a single sample in predicting disability progression. Change in level did not predict any primary outcome in univariable or multivariable analysis, although the HR for conversion to SPMS in a multivariable model of younger patients was relatively large and approached significance. Notably, we assessed changes in sGFAP level over a relatively short period of time (almost 90% of patients had their second sample drawn within 3 years of the first). There is still a role for further research about the relevance of the change in levels over longer periods of time and in specific subpopulations. However, our results suggest that frequent monitoring of sGFAP (e.g., yearly) may not be helpful.

We also found no association between sGFAP levels and future fatigue and cognitive dysfunction in a subset of patients with SDMT and MFIS scores available 2–5 years after biomarkers were drawn. GFAP is correlated with overall brain atrophy and decreased cortical gray matter volume, so it is reasonable to presume an association between GFAP and cognitive dysfunction.^6^, ^15^, ^21^ However, a recent study reported that GFAP does not predict current performance across multiple cognitive domains, including processing speed and verbal memory.^17^ A prior publication from our group also showed no association between sGFAP and future cognitive dysfunction in patients with advanced disability.^16^ Our results extend this previous work, finding no correlation between sGFAP level and future cognitive processing speed regardless of baseline disability. Similarly, while higher EDSS and lower subcortical gray matter volumes are associated with MS fatigue, we found no ability to predict future fatigue using sGFAP levels.^22^, ^23^ To our knowledge, there have been no prior publications on GFAP and fatigue in MS. Given the correlation with GFAP and future imaging outcomes, it could be that our follow‐up interval was too short, and that sGFAP has implications for fatigue and cognition over a longer time period. Alternately, sGFAP may simply not be a good biomarker for fatigue or processing speed. It may be that biomarkers reflecting acute or chronic inflammation, or specific patterns of brain volume loss, are better predictors of future fatigue and cognitive dysfunction.^16^, ^17^, ^22^

As others have shown, sGFAP increased with age, disease duration, and EDSS in our cohort.^5^, ^6^, ^15^ Unlike some studies, we found that disease duration and EDSS remained correlated with sGFAP even when accounting for age. There appeared to be an inflection point in sGFAP levels after roughly age 50, as seen in Figure 1. Notably, four of the five patients excluded as outliers were 57 or older. sGFAP is not specific to MS and can be seen in a variety of neurological conditions that occur more commonly in older patients, including intracerebral hemorrhage and preclinical Alzheimer disease.^24^, ^25^, ^26^, ^27^, ^28^ It may also be that sGFAP levels begin to rise more quickly later in life as part of normal aging.^29^ Consequently, sGFAP should be interpreted cautiously in older MS patients. We also found that sGFAP was higher in women than in men, consistent with prior studies.^6^ Although our results show that sGFAP was higher in patients without new clinical or MRI disease activity in the prior year, this was confounded by older age and more progressive disease in this cohort. Previous publications have reported no impact of MS exacerbations on GFAP levels, and we did not see an effect after accounting for age and other factors.^6^, ^11^

Readers should be aware of other limitations, including the retrospective observational nature of the data. We did not correct for body mass index due to the frequency of missing data in our cohort, although studies have suggested a modest inverse correlation with sGFAP.^6^, ^30^ We also did not convert sGFAP levels to *z*‐scores or age‐normalized values, but instead used multivariable regression models that included age and other covariates such as EDSS. Clinicians should be aware of the importance of interpreting GFAP in the context of age when attempting to apply these results to individual patients. It is worth noting that over 93% of patients had their FSD prior to FDA approval of any anti‐CD20 therapy. While sGFAP was not significantly impacted by treatment in our cohort (as has been shown in other studies), the long‐term impact of high‐efficacy treatment on the trajectory of sGFAP levels is not clear.^13^ Similarly, it may be that early high‐efficacy therapy abrogates the increased risk of disability progression suggested by higher sGFAP levels.

This study is one of the largest analyses of GFAP in MS to date, with 741 patients contributing biomarker data and a median of 10 years of clinical follow‐up. We specifically tried to evaluate a mixture of outcomes relevant to both clinicians and patients, which we believe is a strength of this article. The inclusion of univariable and multivariable proportional hazards models provides data on how sGFAP may be interpreted in the context of known clinical and demographic predictors of disease progression. Several findings are novel, to our knowledge, including the lack of association of sGFAP with fatigue, the limited role for trending sGFAP levels (at least over short intervals), and that sGFAP may help to identify patients at risk of conversion to SPMS and of needing a cane or other gait aid in the future.

## Author Contributions

E.M., B.C.H., N.M., and T.C. contributed to the conception and design of the study. B.C.H., M.P–T., B.I.G., H.L.W., and T.C. contributed to acquisition of the data. E.M., B.C.H., N.M., and M.P–T. contributed to analysis of the data. E.M. drafted the manuscript and prepared the figures. All authors were involved in editing for intellectual content and readability, and in approval of the final version. T.C. takes full responsibility for the data, analysis, and interpretation reported in this study and for the conduct of the research. T.C. has full access to the data and the right to publish any and all data, separate and apart from the guidance of any sponsor.

## Conflict of Interest

T.C. has received consulting fees and research support from Octave, which manufactures a commercial lab test for multiple serum biomarkers, including GFAP.

## Supporting information


Appendix S1.

## Data Availability

Data not provided in the article may be anonymized and shared at the request of any qualified investigator for purposes of replicating procedures and results. To ensure the privacy of participants, access to the data may be subject to approval by the Mass General Brigham Institutional Review Board.
