# Supplementary material for: Serum glial fibrillary acidic protein predicts disease progression in multiple sclerosis
Source: Ann Clin Transl Neurol. 2024 Sep 5;11(10):2719–30. doi: 10.1002/acn3.52187 (PMC11514927; doi:10.1002/acn3.52187)
Supplement: Supplementary file 1 — Appendix S1. [file ACN3-11-2719-s001.docx]

**Supplemental Materials:**

Table A: Characteristics of Excluded Outliers

| Patient | Age | Sex | EDSS | Disease Duration | MS Subtype | sGFAP at FSD (pg/mL) | 2^nd^ sGFAP Level (Years Since FSD) | Selected Co-morbidities |
| --- | --- | --- | --- | --- | --- | --- | --- | --- |
| 1 | 59 | F | 3.5 | 18.5 | RRMS | 2836 | 6932 (1.1) | Depression |
| 2 | 58 | M | 6.5 | 2.0 | PPMS | 3008 | --- | HTN, HLD, remote pontine stroke, recent DVT |
| 3 | 67 | M | 3.5 | 9.0 | SPMS | 1272 | 1459 (1.0) | HTN, HLD |
| 4 | 30 | M | 0 | 2.3 | RRMS | 1345 | 1111 (3.0) | GERD, OSA, migraine |
| 5 | 57 | F | 2.5 | 30.5 | RRMS | 732 | 838 (1.0) | Hypothyroidism, scoliosis |

Summary information for five patients excluded as outliers for abnormally high sGFAP levels. In all cases where a second sample was available, subsequent levels were also extremely high. Patient 5 had a third sample with sGFAP > 1,000 pg/mL. Abbreviations: HTN (hypertension), HLD (hyperlipidemia), DVT (deep vein thrombosis), GERD (gastroesophageal reflux disease), OSA (obstructive sleep apnea)

Table B: Results of Multivariable Linear Regression of sGFAP Level at FSD

| Variable | Coefficient [95% CI] | p-Value |
| --- | --- | --- |
| Age at FSD | 0.777 [0.387 – 1.167] | <0.001 |
| Sex (Male) | -10.790 [-18.623 – -2.958] | 0.007 |
| Race (White) | -4.032 [-16.750 – 8.685] | 0.534 |
| Family History of MS | 0.171 [-7.968 – 8.328] | 0.967 |
| Smoking History | -1.558 [-25.055 – 21.939] | 0.896 |
| EDSS at FSD | 3.309 [0.581 – 6.037] | 0.017 |
| Disease Duration at FSD | 1.062 [0.533 – 1.591] | <0.001 |
| MS Subtype at FSD |  |  |
| RRMS | -8.396 [-24.621 – 7.829] | 0.310 |
| SPMS | 2.258 [-20.380 – 24.897] | 0.845 |
| PPMS | 9.099 [-14.422 – 32.621] | 0.448 |
| Active MS in Prior Year | -5.176 [-12.741 – 2.390] | 0.180 |
| High-Efficacy Treatment at FSD | -7.833 [-19.143 – 3.478] | 0.174 |

Coefficients and p-values from multivariable linear regression to find associations with sGFAP level at FSD (n=741; R-squared=0.193). Age, disease duration, and EDSS were treated as continuous variables. All other variables were treated as categorical (MS subtype reference group: CIS).

Table C: Mean sGFAP Level by Group

| Population | Mean sGFAP [pg/mL] (SD) | p-Value ANOVA |
| --- | --- | --- |
| Sex |  | 0.023 |
| Female (n=520) | 110.3 (52.6) |  |
| Male (n=221) | 100.6 (55.2) |  |
| MS Disease Type |  | <0.001 |
| CIS (n=39) | 101.2 (73.8) |  |
| RRMS (n=604) | 102.0 (47.6) |  |
| PPMS (n=38) | 138.8 (71.8) |  |
| SPMS (n=60) | 146.0 (59.7) |  |
| Race |  | 0.997 |
| White (n=678) | 107.4 (52.6) |  |
| Non-White (n=63) | 107.4 (63.3) |  |
| Family History of MS |  | 0.509 |
| Yes (n=206) | 110.6 (54.9) |  |
| No (n=445) | 105.6 (51.1) |  |
| Smoking History |  | 0.570 |
| Yes (n=302) | 109.1 (59.3) |  |
| No (n=421) | 105.9 (48.6) |  |
| New MS Activity in Past Year |  | <0.001 |
| Yes (n=321) | 97.1 (45.9) |  |
| No (n=420) | 115.3 (57.6) |  |
| Treatment at FSD |  | 0.094 |
| High Efficacy (n=92) | 102.3 (42.9) |  |
| Moderate Efficacy (n=465) | 106.0 (51.0) |  |
| Untreated (n=19) | 132.8 (63.5) |  |

Table of mean sGFAP levels for each categorical variable. Patients without information on family history or other characteristics were omitted from the relevant row. Therefore, not all totals sum to 741.

Table D: Rate of Primary Outcomes

| Outcome | Number of Patients Reaching Outcome |
| --- | --- |
| PIRA | 374 (50.5%) |
| Future Gait Aid | 138 (18.6%) |
| Conversion to SPMS | 102 (15.9%) |

The number of patients reaching each primary outcome. PIRA and future gait aid requirement were assessed for all 741 patients. Conversion to SPMS was assessed for the 643 patients without progressive disease at first sGFAP sample.

Figure A: Baseline sGFAP Levels

Histogram of sGFAP levels at FSD for all 741 patients included in the analysis. Descriptive statistics: mean: 107.5, standard deviation: 53.4, median: 95.6, skewness: 1.8, kurtosis: 8.9.

Figure B: sGFAP Level by Disease Activity and Treatment Status

1. sGFAP levels among those with and without new clinical or MRI disease activity in the year prior to FSD. Notably, the difference in sGFAP level by disease activity was mediated by older age and more progressive disease in the inactive group. *(B)* sGFAP graphed against treatment status at FSD (MET: moderate-efficacy therapy; HET: high-efficacy therapy). There was no significant difference in sGFAP level by ANOVA.
